# Gremlin, a Bone Morphogenetic Protein Antagonist, Is a Crucial Angiogenic Factor in Pituitary Adenoma

**DOI:** 10.1155/2015/834137

**Published:** 2015-03-05

**Authors:** Kenta Koketsu, Daizo Yoshida, Kyongsong Kim, Yudo Ishii, Shigeyuki Tahara, Akira Teramoto, Akio Morita

**Affiliations:** Department of Neurosurgery, Nippon Medical School, Tokyo 113-8602, Japan

## Abstract

Gremlin is an antagonist of bone morphogenetic protein (BMP) and a major driving force in skeletal modeling in the fetal stage. Several recent reports have shown that Gremlin is also involved in angiogenesis of lung cancer and diabetic retinopathy. The purpose of this study was to investigate the role of Gremlin in tumor angiogenesis in pituitary adenoma. Double fluorescence immunohistochemistry of Gremlin and CD34 was performed in pituitary adenoma tissues obtained during transsphenoidal surgery in 45 cases (7 PRLoma, 17 GHoma, 2 ACTHoma, and 2 TSHoma). Gremlin and microvascular density (MVD) were detected by double-immunofluorescence microscopy in CD34-positive vessels from tissue microarray analysis of 60 cases of pituitary adenomas (6 PRLoma, 23 GHoma, 22 NFoma, 5 ACTHoma, and 4 TSHoma). In tissue microarray analysis, MVD was significantly correlated with an increased Gremlin level (linear regression: *P* < 0.005,  *r* ^2^ = 0.4958). In contrast, Gremlin expression showed no correlation with tumor subtype or Knosp score. The high level of expression of Gremlin in pituitary adenoma tissue with many CD34-positive vessels and the strong coherence of these regions indicate that Gremlin is associated with angiogenesis in pituitary adenoma cells.

## 1. Introduction

Angiogenesis is a complex multistep process that has a crucial role in tumor growth, invasion, and metastasis. Improved understanding of angiogenesis will provide insights into tumor stage and the response of tumor vessels to antiangiogenic therapy and may lead to more personalized cancer therapy [1, 2]. Current attempts to disrupt tumor blood vessel formation are predominantly focused on targeting the VEGF-VEGFR signaling pathway [2]. Pituitary tumors are highly vascular neoplasms, which suggests an important role of angiogenesis in pituitary tumor growth, but the mechanisms that underlie tumorigenesis in pituitary adenomas are uncertain [3, 4]. In particular, the mechanism that controls tumor angiogenesis and whether this process is required for tumor growth have been the subject of much discussion.

Microvascular density (MVD) has been studied in a number of neoplasia and generally there is a close relationship between angiogenesis and tumor progression. Thus, MVD may be a predictive factor for disease progression and response to treatment. Zhang et al. found that cervical cancer progression is correlated with MVD and VEGF [5], and Zhao et al. showed that VEGF and MVD are decreased by siRNA silencing of c-Src, a predictor of a poor prognosis in pancreas cancer [6]. Norcantharidin, an angiogenic inhibitor in gallbladder cancer, has been shown to inhibit cancer cell proliferation, migration, and invasion and to reduce angiogenesis based on decreased MVD and VEGF expression [7].

VEGF is likely to have a role in tumor angiogenesis in pituitary adenomas that is similar to that in other neoplasms, and VEGF also regulates the growth of pituitary tumor cells through its receptors VEGFR-1, VEGFR-8, and VEGFR-9. Onofri et al. [8, 9] showed that ligands of VEGF receptors influence angiogenesis in pituitary adenomas and affect growth of pituitary tumor cells through VEGFR-1, and that VEGF and VEGFR-3 immunostaining in pituitary tumors was higher than in normal pituitary tissue. These results indicate that the VEGF-C/VEGFR-3 system might be involved in controlling tumor angiogenesis in pituitary adenomas lacking lymphatic vessels and may also play a role in initiating tumor lymphangiogenesis. Horiguchi et al. [10] showed that transforming growth factor (TGF) *β*1 may regulate angiogenesis in pituitary adenomas by initially increasing levels of proangiogenic VEGF-A and then stimulating antiangiogenic molecules in a human pituitary cell line (HP75). Zatelli et al. [11] showed that somatostatin exerts antiproliferative effects by inhibiting VEGF secretion and action and that VEGF expression may be related to pituitary tumor growth. Kim et al. reported that knockout of the pituitary tumor-transforming gene (PTTG), which is associated with tumorigenesis, decreases proliferation, vascular invasion, and proangiogenic factors, including fibroblast growth factor (FGF2) and its receptor FGFR1 and VEGF, in mouse thyroid cancer with PTTG overexpression [12]. A novel PTTG-mediated proliferative pathway may be critical in thyroid cancer growth and progression by upregulating VEGF and kinase insert domain receptor (KDR) expression [13, 14]; however, in contrast, it has been shown that VEGF is not correlated with MVD [15]. Thus, it remains unclear whether VEGF is critically involved in regulating tumor angiogenesis in pituitary adenomas.

Gremlin is an antagonist of bone morphogenic protein (BMP) and is expressed during embryonic development and under different pathologic conditions, including cancer. Gremlin was identified as a novel proangiogenic factor belonging to the cysteine-knot superfamily that includes TGF-*β* and VEGF. Gremlin is expressed in the endothelium and stimulates migration and invasion of endothelial cells in fibrin and collagen gels, binds with high affinity to various endothelial cells, and triggers tyrosine phosphorylation of intracellular signaling proteins [16]. Similarly to VEGF, Gremlin activates VEGFR2 in endothelial cells, leading to VEGFR2-dependent angiogenic responses* in vitro* and* in vivo* [17, 18]. Thus, Gremlin is a novel proangiogenic VEGFR2 agonist that is distinct from VEGF family ligands and has implications in vascular development and tumor neovascularization [18, 19].

To the best of our knowledge, expression of Gremlin has not been examined in pituitary adenomas. Therefore, the aim of this study was to investigate the role of Gremlin in tumor angiogenesis in pituitary adenomas. Our results show a close relationship of Gremlin with tumor angiogenesis and proliferation in human pituitary adenoma tissues.

## 2. Material and Methods

### 2.1. Double-Fluorescence Immunohistochemistry

Forty-five pituitary adenoma tissues samples were selected from operative specimens obtained during transsphenoidal surgery in the Department of Neurosurgery at Nippon Medical School from April 2010 to August 2011. The subjects included 28 women (17–76 years old) and 17 men (22–75 years old). Based on previous immunohistochemical staining data, tumors were classified as ACTHoma (*n* = 2), GHoma (*n* = 17), NFoma (*n* = 17), PRLoma (*n* = 7), and TSHoma (*n* = 2). No patients with acromegaly received octreotide and none with prolactinoma received preoperative dopamine antagonists.

All specimens were promptly fixed in 10% buffered formalin, embedded in paraffin, and stored. After characterization for pituitary hormones, 4 *μ*m sections of slide-mounted paraffin blocks were stained for double-immunofluorescence detection of CD34 and Gremlin. After routine deparaffinization, slides were placed in a glass jar filled with target retrieval solution (pH 6.0; Dako Real, DakoCytomation) and boiled for 5 min in a microwave oven at 600 W to retrieve antigen. Slides were cooled at room temperature for 30 min, rinsed in phosphate-buffered saline (PBS), and blocked by incubation with 1% nonfat milk (Block Ace, Dainippon Pharmaceutical Co., Ltd., Tokyo, Japan) for 30 min. Slides were then incubated with mouse anti-human CD34 monoclonal antibody (0.05 mol/L; Dako North America, Inc., Carpinteria, CA) for 30 min followed by incubation with Texas Red- (TR-) conjugated goat anti-mouse IgG (1 : 100; Santa Cruz, Inc., Santa Cruz, CA) for 30 min. Thereafter, sections were incubated with rabbit anti-human Gremlin polyclonal IgG antibody (1 : 200; Santa Cruz) for 60 min followed by incubation with FITC-conjugated bovine anti-rabbit IgG (1 : 100; Santa Cruz) for 60 min. After counterstaining with Meyer's hematoxylin, each section was mounted with mounting medium (Gel/Mount, Biomeda Corp, Foster City, CA). Expression in each section was evaluated in six randomly selected visual fields by fluorescence microscopy (Olympus BX-51, Olympus, Tokyo, Japan) at 40x magnification and analyzed using a computerized image analysis system (Image-Pro Plus ver. 4.5, Media Cybernetics, Silver Spring, MD). Bitmap analysis was used to examine the distribution of the luminance of regions stained in fluorescence immunohistochemistry using Image-Pro Plus, with quantification based on numerical brightness values.

### 2.2. Tissue Microarray Analysis to Detect Gremlin in Pituitary Adenoma Tissue

Pituitary adenoma tissues samples were selected from 60 subjects, including 28 women (17–76 years old) and 32 men (22–75 years old) with ACTHoma (*n* = 5), GHoma (*n* = 23), NFoma (*n* = 22), PRLoma (*n* = 6), and TSHoma (*n* = 4). Samples were paraffin embedded and used to build tissue microarrays that were analyzed immunohistochemically using a protocol available online (http://genome-www.stanford.edu/TMA/). Tissue microarrays were incubated with rabbit anti-human Gremlin polyclonal antibody (1 : 100 dilution), rabbit anti-*β*-actin monoclonal antibody (positive control; 1 : 100 dilution), or normal rabbit IgG (negative control; 0.1 lg/mL), followed by incubation with a secondary antibody (1 : 100 dilution; anti-rabbit IgG-FITC; all antibodies are from Santa Cruz Biotechnology, Santa Cruz, CA). Expression was examined by fluorescence microscopy (FMV 1000; Olympus, Tokyo, Japan). The fluorescence intensity of the negative control was subtracted and the level of Gremlin relative to *β*-actin was calculated as a percentage using image analysis software (Image Pro-Plus, ver. 6.3; Media Cybernetics Division of Nippon Roper, Tokyo, Japan).

### 2.3. Statistical Analysis

Relationships between CD34-positive vessels and Gremlin expression in pituitary adenoma tissues were evaluated using quadratic regression analysis. Correlations between the Knosp score, a measure of pituitary tumor invasiveness, and Gremlin expression (relative to *β*-actin) in microarray analyses were analyzed by Spearman rank correlation test. Comparisons between microadenomas (<1 cm) and macroadenomas (>1 cm) in preoperative images (MRI enhanced with Gd-DTPA) and tissue microarray analysis were analyzed by Mann-Whitney test. A Kruskal-Wallis test followed by a post hoc Dunn multiple comparison test was used for group comparisons of Gremlin among pituitary adenoma subtypes. The relationship between CD34-positive vessels and Gremlin expression determined by tissue microarray analysis was evaluated using linear regression analysis. All statistical analyses were performed using GraphPad Prism ver. 5.0 (GraphPad Software, CA, USA). *P* ≤ 0.05 was considered significant. All data are shown as means ± standard deviation (SD).

## 3. Results

### 3.1. Double-Fluorescence Immunohistochemistry

**Figure 1:**
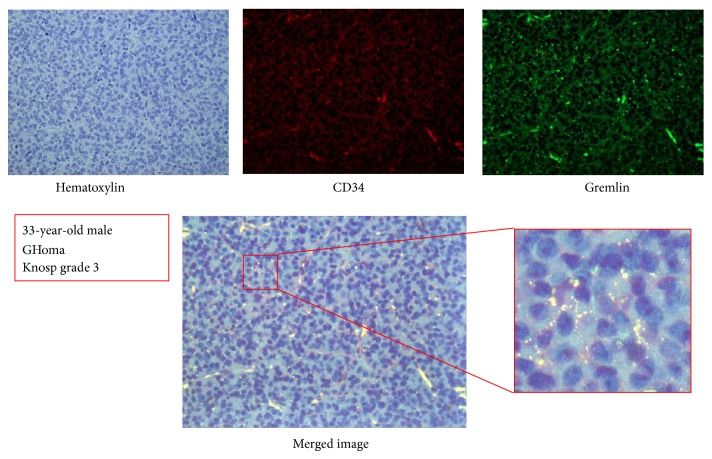
Merged double immunofluorescence image. Expression of Gremlin and CD34 in pituitary adenoma tissue in a representative case of a 33-year-old male with GHoma, Knosp grade 3; 40x magnification. Yellowish fluorescence indicates colocalization of Gremlin and CD34 in cytoplasma of tumor parenchymal cells (Gremlin, FITC; CD34, PI).

Double-fluorescence immunohistochemistry revealed that Gremlin is present in various subtypes of pituitary adenomas. Localization of Gremlin is mainly cytoplasma in tumor parenchymal cells. A representative image from the case of a 33-year-old male with GHoma, Knosp grade 3 is shown in Figure 1. Using the image analysis software (Image Pro-Plus ver. 7.0), presence of Gremlin was quantified, the intensity of the fluorescent probes was measured, and the sum of the points that are fluorescent above a unified brightness was calculated by pixel. MVD which corresponds to the number of CD34-positive vessels was also measured by the same method in the same visual field.

**Figure 2:**
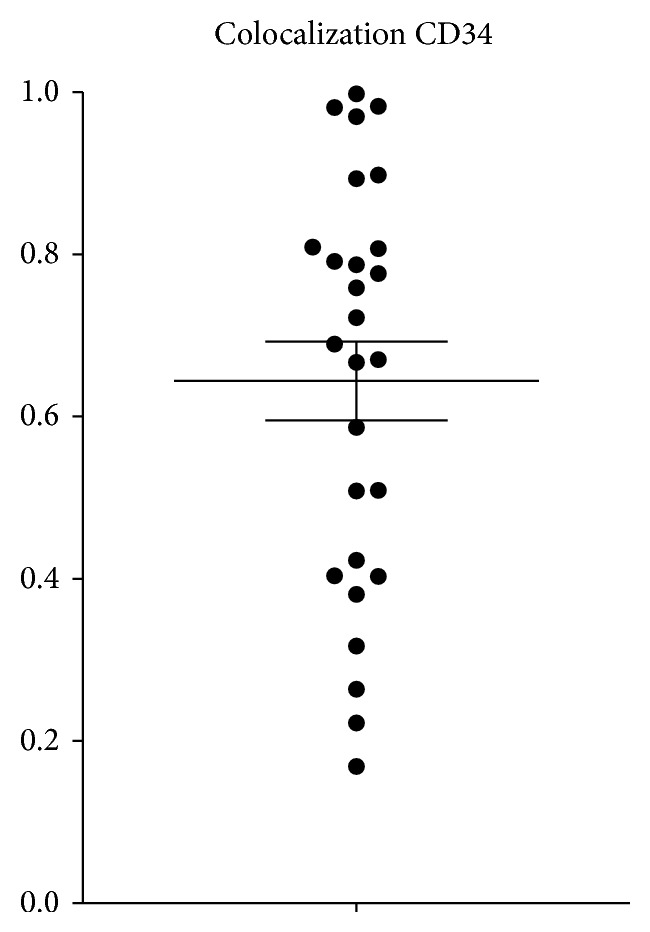
The rate of colocalization with Gremlin in CD34-positive vessels ranged from 0.169 to 0.998 (mean 0.644, SEM 0.049).

Merged images from double-fluorescence immunohistochemistry in tissue samples showed colocalization of Gremlin and CD34 in the vascular endothelium. Using the colocalization analysis tool image analysis software (Image Pro-Plus ver. 7.0), the area of the region of overlapping fluorescent probe was calculated by pixel. The rate of colocalization with Gremlin is in CD34-positive cells in the range of 0.169 to 0.998; the average is 0.644 (64.4%) (SEM 0.049) (Figure 2). Gremlin and CD34-positive cells were shown to exist with most in the equivalence place.

### 3.2. Tissue Microarray Analysis

**Figure 3:**
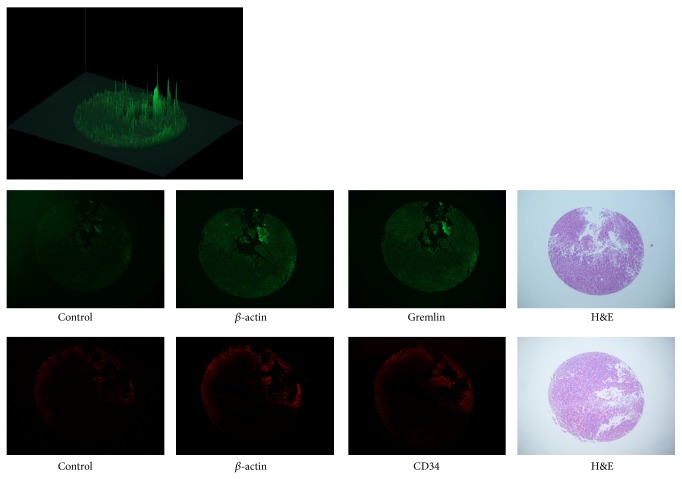
Brightness was quantified by image analysis software (Image Pro-Plus ver. 7.0) was measured (upper left). Tissue microarray analysis of 60 cases. *β*-actin was used as a positive control and normal rabbit IgG as a negative control (Gremlin, FITC; CD34, PI).

**Figure 4:**
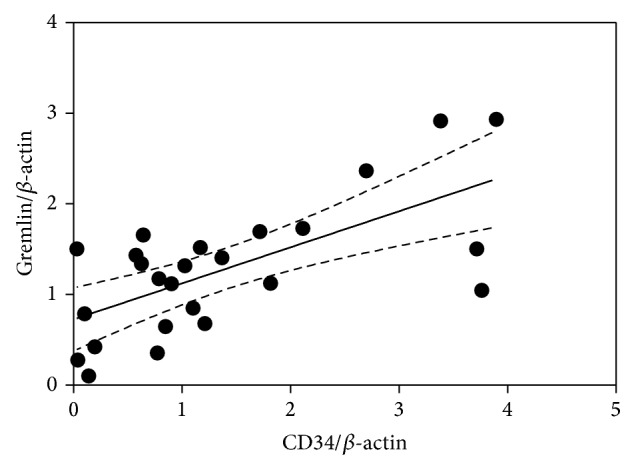
Linear regression analysis of tissue microarray data. Normalized Gremlin and CD34 levels were significantly correlated (*P* < 0.005; 95% confidence interval: 0.025–0.042; *r* ^2^ = 0.4958; *F* = 32.24).

Tissue microarray analysis of 60 pituitary adenomas was performed with the goal of detecting Gremlin expression in tumor subtypes, using *β*-actin as a positive control and normal rabbit IgG as a negative control (Figure 3). Brightness was quantified by image analysis software (Image Pro-Plus ver. 7.0) was measured (Figure 3 upper left). Linear regression analysis of tissue microarray analysis data proved that an existence level of gremlin and CD34 significantly showed equilateral correlation (*P* < 0.005; 95% confident interval: 0.025–0.042; *r* ^2^ = 0.4958; *F* = 32.24) (Figure 4). Neither Gremlin nor CD34 expression showed a significant relationship with tumor subtypes, Knosp score (evaluation score of the degree of infiltration into the cavernous sinus of pituitary adenoma), tumor size, sex, or age (data not shown).

## 4. Discussion

In the current study, Gremlin was shown to be expressed abundantly in pituitary adenoma tissues. This expression was significantly related to CD34-positive vessels, but not to the tumor invasion grade or age, sex, or tumor size. These results strongly indicate that Gremlin may regulate tumor angiogenesis.

Gremlin is a glycoprotein that is expressed during embryonic development and acts as a BMP agonist by binding to BMPs 2, 4, and 7. Gremlin is expressed in osteoblasts and opposes BMP effects on osteoblastic differentiation and function. Head muscle formation is locally repressed by Wnt and BMP signaling and induced by antagonists of these signaling pathways secreted by adjacent tissues [20]. Gremlin has been shown to play a role in dorsal-ventral patterning, in tissue remodeling, and recently in angiogenesis. Members of the bone morphogenetic protein (BMP) family are growth factors known to play a key role in vascular development. In Matrigel assays, BMP modulators chordin and noggin had no stimulatory effect; however, Gremlin and Tsg enhanced human umbilical vein endothelial cell (HUVEC) sprouting [21]. Gremlin expression is significantly increased in lung adenocarcinoma samples compared to matched normal tissues. Gremlin increases cell growth through a BMP-independent pathway [22]. Gremlin expression was significantly associated with high MVD. MVD was significantly higher in well-differentiated pancreatic neuroendocrine tumors than in well-differentiated or poorly differentiated neuroendocrine carcinomas [23]. Transgenic mice overexpressing Gremlin in the bone microenvironment have decreased osteoblast number and function, leading to osteopenia and spontaneous fractures [24]. Initiation of metanephric kidney development requires reduction of BMP4 activity by Gremlin in the mesenchyme, which in turn enables ureteric bud outgrowth and establishment of autoregulatory GDNF/WNT11 feedback signaling [25]. BMPs are synthesized in skeletal cells and deletion of Gremlin in the skeleton results in increased BMP signaling and activity. This increases trabecular bone volume due to elevated osteoblastic activity and increased mineral apposition and bone formation, without changes in osteoblast number and bone resorption [26]. Genetos et al. [27] also found that hypoxia decreases sclerostin expression through enhanced antagonism of BMP signaling, independent of VEGF.

Gremlin is also expressed under pathological conditions, including in cancer, and is a proangiogenic protein in the cysteine-knot superfamily that also includes TGF-*β* and VEGF. Gremlin activates VEGFR2 in endothelial cells, leading to VEGFR2-dependent angiogenic responses with implications in vascular development, angiogenesis-dependent diseases, and tumor neovascularization [28]. Gremlin is also widely expressed in cancer-associated stromal cells in basal cell carcinoma of the skin, a common human cancer, and BMP antagonists may be important constituents of tumor stroma, providing a favorable microenvironment for cell survival and expansion in many cancers [29].

Abundant Gremlin expression also occurs in diabetic nephropathy [30, 31], occasionally in glomeruli, but most prominently in areas of tubulointerstitial fibrosis, where it colocalizes with TGF-*β* and is directly correlated with renal dysfunction [32]. BMP-7 and Gremlin are involved in renal development and diabetic nephropathy and undergo expression changes in the diabetic kidney [33, 34]. Dolan and colleagues proposed that reactivation of Gremlin (and BMP-7) in the diabetic kidney is a novel therapeutic target for diabetic nephropathy, since administration of the Gremlin ligand BMP-7 is protective in models of progressive renal diseases [30]. This function of Gremlin in diabetic nephropathy is an example of reemergence of developmental programs in disease and indicates that Gremlin warrants attention in the context of developmental nephrology [31].

Gremlin expression is also induced in bovine renal pericytes in response to elevated glucose and in the retina of the streptozotocin-induced diabetic mouse. This expression is modulated by hyperglycemic induction of MAPK, reactive oxygen species, and TGF-*β* pathways, all of which have a role in diabetic fibrotic disease. This implies a role for Gremlin in the pathogenesis of diabetic retinopathy [35]. Gremlin binds VEGFR2, the main transducer of VEGF-mediated angiogenic signals, in a BMP-independent manner. Similarly to VEGF-A, Gremlin activates VEGFR2 in endothelial cells, leading to VEGFR2-dependent angiogenic responses [18]. Thus, Gremlin is a novel proangiogenic VEGFR2 agonist that is distinct from VEGF family ligands and has implications in vascular development and tumor angiogenesis. Gremlin may also have a role in regulating self-renewing tumor cell compartments [6].

One of the few studies on Wnt signaling in normal pituitary or adenoma cells revealed a critical function of the Wnt pathway in control of the progenitor/stem cell pool in the pituitary and, in particular, in craniopharyngioma [36]. Giles et al. showed that Wnt4 is expressed in the adult pituitary gland and that its expression is increased by estrogen exposure, suggesting that adult tissue plasticity is likely to involve *β*-catenin-independent signaling pathways, and conclusively showed Wnt signaling in estrogen-induced lactotroph proliferation [37]. A review of 13 whole genome approaches in pituitary tumors with the goal of identifying Wnt family inhibitors showed that expression of WF1, SFRP2, FRZB, SFRP4, DKK2, and SOSTDC1 genes is decreased in pituitary adenomas compared to normal pituitary tissue, while that of SFRP1 and SFRP4 is increased [38].

In the current study, we found that Gremlin is strongly expressed in pituitary adenoma tissues and that the expression level was significantly associated with CD34-positive vessels. These data suggest the possibility that Gremlin plays an important role by the tumor angiogenesis in pituitary adenomas. Signaling cascades mediated by Gremlin should be further investigated since Gremlin may be a novel candidate for molecular targeted therapy for pituitary adenoma.
